# Experimental Study on the Mechanical Properties and Durability of High-Content Hybrid Fiber–Polymer Concrete

**DOI:** 10.3390/ma14216234

**Published:** 2021-10-20

**Authors:** Chaohua Zhao, Zhijian Yi, Weiwei Wu, Zhiwei Zhu, Yi Peng, Jie Liu

**Affiliations:** School of Civil Engineering, Chongqing Jiaotong University, Chongqing 400074, China; Yizj63@126.com (Z.Y.); www92@whut.edu.cn (W.W.); 15223155792@163.com (Z.Z.); py.peng@outlook.com (Y.P.); lj345741251@163.com (J.L.)

**Keywords:** steel fiber-reinforced concrete, polymer-modified concrete, crack resistance mechanism, mechanical properties, durability

## Abstract

Polymer-modified concrete and fiber concrete are two excellent paving materials that improve the performance of some concrete, but the performance of single application material is still limited. In this paper, polymer-modified concrete with strong deformation and fiber concrete with obvious crack resistance and reinforcement effect were compounded by using the idea of composite material design so as to obtain a high-performance pavement material. The basic mechanical properties of high-content hybrid fiber–polymer-modified concrete, such as workability, compression, flexural resistance, and environmental durability (such as sulfate resistance) were studied by using the test regulations of cement concrete in China. The main results were as follows. (1) The hybrid fiber–polymer concrete displayed reliable working performance, high stiffness, and a modulus of elasticity as high as 35.93 GPa. (2) The hybrid fiber–polymer concrete had a compressive strength of 52.82 MPa, which was 31.2% higher than that of the plain C40 concrete (40.25 MPa); the strength of bending of the hybrid concrete was 11.51 MPa, 191.4% higher than that of the plain concrete (3.95 MPa). (3) The corrosion resistance value of the hybrid fiber–polymer concrete was 81.31%, indicating its adjustability to sulfate attack environments. (4) According to cross-sectional scanning electron microscope (SEM) images, the hybrid fiber–polymer concrete was seemingly more integrated with a dense layer of cementing substance on its surface along with fewer microholes and microcracks as when compared to the ordinary concrete. The research showed that hybrid fiber–polymer concrete had superior strength and environmental erosion resistance and was a pavement material with superior mechanical properties.

## 1. Introduction

Polymer-modified cement concrete is a composite material formed by the addition of water-soluble organic polymer to cement concrete. The polymer demulsifies within the concrete structure to form a film and fills in the concrete voids, forming a structural network with the hydration products of the cement, which in turn alters the internal concrete microstructure. When compared with ordinary cement concrete, the deformation performance, bending resistance, durability, and impermeability are improved [1,2,3,4,5]. From a microscopic perspective, polymer emulsion forms films internally during the curing process for the formation of strength; the films fill the gaps between the coarse and fine aggregates, enhance the bonding effect of the aggregates, achieve a better density, and (to a certain extent) improve the shrinkage deformation of the concrete.

Steel fiber-reinforced concrete can significantly improve the bending-tensile performance, crack resistance, durability, and fatigue performance of the component structures [6,7,8,9,10,11] and has thus been utilized in many fields of civil engineering. Poor performance is often observed when normal shotcrete supports are used in the rock surrounding the tunnels because of the large deformation of the surrounding rock and insufficient strength and deformability of the concrete. In countries such as Norway, steel fiber shotcretes and anchors are used as reinforcements for tunnel support [12,13,14]. Steel fiber-reinforced concrete is also used for roof waterproofing, where its main advantages are favorable temperature stability, high impermeability, and superior crack resistance. However, because of the influence of construction workability and uniformity, the volume content of FRC containing just a single kind of ordinary steel fiber is less than 3%, and the role of the fiber is relatively limited.

The use of fibers as mass reinforcement to delay cracking and to improve the strength and the postcracking performance of reinforced concrete (RC) beams has been well documented [15]. Ultra-high-performance concrete (UHPC) is a rapidly emerging concrete material that has an ultrahigh compressive strength and bond strength. When it is reinforced with short, discontinuous fibers, it features a tensile strain-hardening behavior and a damage pattern of closely spaced narrow cracks [16].The literature shows that when the content of steel fiber is less than 3%, the strength of ordinary steel fiber mortar is slightly higher than that of shear ultrashort ultrafine steel fiber mortar. In contrast, when the steel fiber content is greater than 3%, the strength of shear ultrashort ultrafine steel fiber mortar is significantly higher than that of ordinary steel fiber mortar. Ordinary steel fiber concrete specimens still have a certain residual strength after cracking. Ultrashort ultrafine steel fiber can more effectively improve the compressive performance of concrete, and hybrid steel fiber can improve the toughness of concrete to a great extent. High content of ordinary steel fiber, however, brings the problem of uneven distribution. Therefore, ordinary steel fiber-reinforced concrete aims to improve the performance of concrete after cracking, and ultrashort and ultrafine steel fiber-reinforced concrete aims to improve overall compressive performance and toughness. Based on the fracture mechanics mechanism of fiber-reinforced concrete, flexible fiber is added mainly to solve the shrinkage problem in the solidification process.

Compared to ordinary concrete, polymer-modified cement concrete has shown better deformability, cohesion, durability, wear resistance, and impermeability and is widely used. However, it is burdened by high polymer price, relatively complex construction process, and slightly insufficient mechanical indexes in areas such as bending-tensile strength, impact resistance, and toughness when used for certain special pavements. Steel fiber-reinforced concrete, on the other hand, shows excellent toughness, bending-tensile resistance, impact resistance, fatigue resistance, and durability, but has inadequate adhesion between the steel fiber and the concrete (i.e., the steel fiber tends to get pulled out rather than being broken during a failure). Therefore, steel fiber-reinforced concrete is not utilized to its full potential. In addition, its deformation performance and wear resistance remain insufficient for special paving [17,18,19,20,21,22,23,24]. To maximize the advantages of the two materials and overcome shortcomings, they were combined to form the modified hybrid fiber–polymer cement concrete. Polymer-modified cement concrete resolves the problems of penetration, wear resistance, sliding resistance, deformation, initial cracking, and shrinkage. In contrast, the steel fiber reinforced concrete covers the issues of bending-tensile resistance, shear resistance, impact resistance, and fatigue resistance [25]. The idea of composite materials design was applied to make full use of the advantages of the two materials and to reflect the aforementioned synergistic effect.

This study presents the author’s systematic results. A large number of experiments were carried out in the early stage. The aim of this research was to create high-content fiber concrete with uniform dispersion. The uniformity of fiber concrete directly affects the performance of materials. In order to achieve uniform distribution, polymer materials were introduced in this work. The addition of polymer materials actually enabled the uniform distribution of high content fibers. Based on previous exploration, this research found that the addition of some polymers can significantly change the workability of concrete so as to improve the maximum content of fiber. Therefore, in this study, hybrid fiber–polymer concrete with high fiber content was created. When polymer emulsion and steel fiber of a certain proportion were mixed into the concrete, the emulsion formed a network structure on the surface of the steel fiber and the aggregates of the mixing process, which reduced the concentration of stress and enhanced the adhesion between the steel fiber and the aggregate. Therefore, the properties of toughness, density, crack resistance, and fatigue resistance for the whole material were improved.

The material studied in this paper had high mechanical properties and durability. It is expected to be used on special occasions with high performance requirements, such as steel bridge deck pavement, factory roads in harsh environments, etc.

## 2. Materials and Test Methods

### 2.1. Raw Materials and Parameters

The characteristic parameters and sources of procurement of the raw materials were as follows.

**Figure 1:**
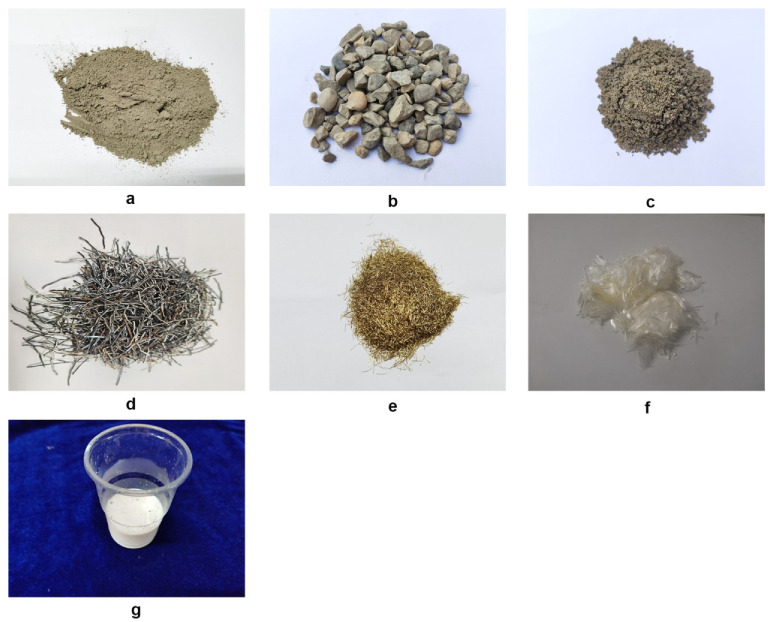
Raw materials: (**a**), ordinary 42.5 silicate type Huaxin Cement; (**b**), cobblestones with a maximum nominal grain size of 10 mm and a grading of 5–10 mm; (**c**), fly ash; (**d**), ordinary steel fiber; (**e**), ultrashort ultrafine steel fiber; (**f**), polypropylene flexible fiber; (**g**), polymer emulsion.

Cement: Ordinary 42.5 silicate type Huaxin Cement produced in Chongqing, as shown in Figure 1a.

Gravel: Locally produced cobblestones from Chongqing with a maximum nominal grain size of 10 mm and a grading of 5–10 mm, as shown in Figure 1b.

Fly ash: Grade I fly ash locally produced in Chongqing, as shown in Figure 1c.

Ordinary steel fiber: Sheared wavy steel fiber (produced by Liaoning Anshan Kebite Technology Co., Ltd., Anshan City, Liaoning province, China.) with length of 35 mm and diameter of 0.87 mm, as shown in Figure 1d.

Ultrashort ultrafine steel fiber: Melted and drawn ultrashort ultrafine steel fiber manufactured by Liaoning Anshan Kebite Technology Co., Ltd. with length of 6 mm and diameter of 0.2 mm, as shown in Figure 1e.

Flexible fiber: Polypropylene flexible fiber with length of 12 mm (manufactured by Langfang Haoxin Company of Thermal Insulation, Fireproofing, and Sealing Materials Co., Ltd., Langfang City, Shandong province, China.), as shown in Figure 1f.

Polymer emulsion: Independently developed polymerized compound emulsion, as shown in Figure 1g.

Table 1 provides a summary of the basic physical and mechanical parameters of the sheared wavy steel fiber, the ultrashort ultrafine steel fiber, and the flexible fiber.

### 2.2. Mix Proportion Design

After the preparation of the trials, the ratios of volume of the ordinary steel fiber, the ultrashort ultrafine steel fiber, and the flexible fiber (polypropylene fiber) were determined to be 1.92%, 4.00%, and 0.27%, respectively. The unit cubic mass mix proportion used in this study was as follows: cement (42.5 ordinary silicate cement)/fly ash/gravel (granite)/medium–coarse sand/ordinary steel fiber/ultrashort ultrafine steel fiber/polypropylene fiber/polymer emulsion = 580:250:600:600:150:312:3:320 kg.

### 2.3. Test Method

#### 2.3.1. Slump Test

Slump is mainly used to characterize the plasticizing and pumpable performance of concrete, and the requirements for application to different structures are different. This paper aimed to investigate a material for application to some special pavement structures, such as steel bridge decks, to be used mainly by paving. Therefore, the smaller the slump, the better the paving effect and material performance were judged to be.

The slump test detects the water retention, fluidity, and cohesion of the concrete matrix and also verifies the feasibility and workability of the designed mix proportion. According to the Standard for the Test Method of Performance on Ordinary Fresh Concrete (GB/T50080-2016) [26], the following measures were taken during the slump test.

(1) The inside of the slump barrel was moistened with water to prevent the adhesion of the concrete mixture with the inner barrel wall, which could potentially cause bonding damage to the concrete during the falling off process. Next, the barrel was placed on a dry, horizontal plate made of steel or wood to avoid the influence of plate inclination and humidity on the test results. The slump barrel was fixed during the filling process, and both feet were stepped on the pedal for loading.

(2) When the materials were loaded to 1/3 and 2/3 of the barrel height, the tamping rod was used for continuous and stable tamping around 20 to 30 times. The tamping rod penetrated the concrete composite matrix without causing an impact to avoid the dispersion of the composite. When the materials were loaded to the top of the slump barrel, then the excess mixture was scraped off with a trowel, and the top surface was smoothened with a scraper.

(3) After the excess concrete materials around the bottom of the barrel were removed, the barrel was lifted within 5 to 10 s. During this period, the slump barrel was lifted vertically with a uniform speed without imposing transverse or twisting forces on the concrete. The whole process from the beginning of loading to the lifting of the slump barrel was completed within 150 s.

(4) A flat plate, which came in contact with the concrete mixture but did not exert force, was gently placed on the top of the concrete mixture during the slump measurement process. The height h was measured from the plate to the bottom with a measuring scale, and then h was subtracted from the height H of the slump barrel. The result obtained was the measured slump, expressed in the unit of mm.

**Figure 2:**
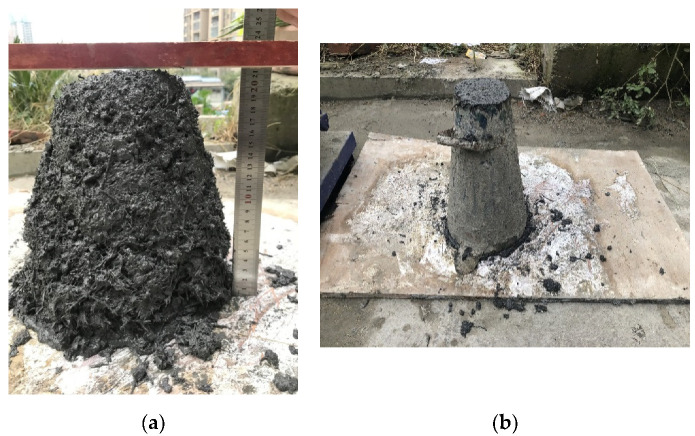
Measurement processes in the slump test: (**a**) measuring; (**b**) forming.

In the slump test, four basic processes were generally included in the preparation of the concrete mixture: weighing, mixing, loading, and measuring. The measuring is shown in Figure 2.

#### 2.3.2. Elasticity Modulus Test

**Figure 3:**
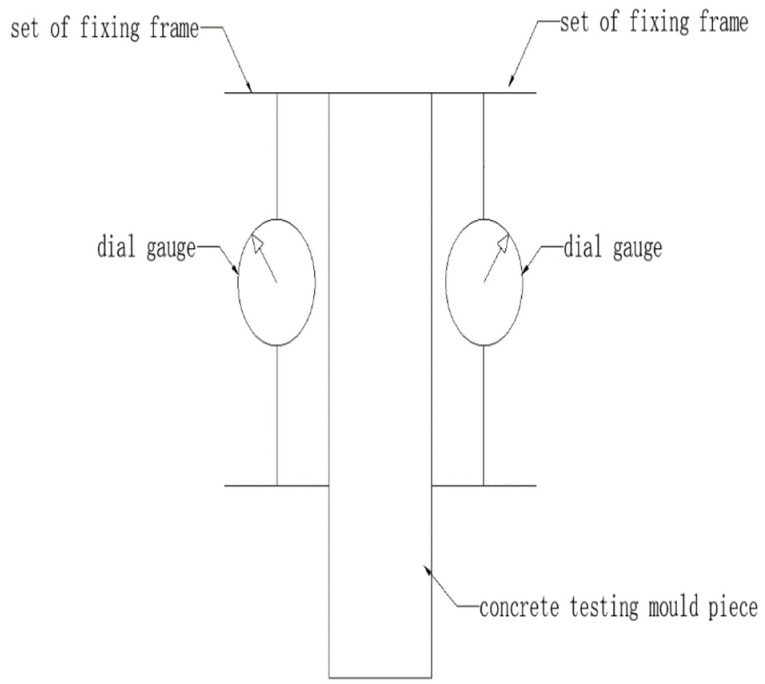
Sketch map of the elasticity modulus loading test.

The test method for the elasticity modulus under static pressure was performed as specified in *Standard for Test Method of Mechanical Properties on Ordinary Concrete* (GB/T 50081-2002) [27]. The test apparatuses, as shown in Figure 3, consisted of one universal testing machine, one ball socket, two dial gauges, two sets of fixing frames for the measuring instrument, and one steel ruler. In the test, the sample size was 150 mm × 150 mm × 300 mm. Two groups of three samples were used. The first group was used for conducting the static compression test to determine the compressive strength and thus the loading basis of the elasticity modulus test. The second group was used for the formal elasticity modulus test.

During the elasticity module test, the two dial gauges were installed on the fixed frame and symmetrically arranged on both sides of the sample, and the bottom of the dial gauge was close to the centerline on both sides of the test piece. The sample was placed on the central region of the platform, and the press machine was turned on to gradually lower the upper platform and ensure its balanced contact with the sample.

The sample was pretested before the formal test. The loading principle was as follows. First, initial stress of 0.5 MPa was applied, which corresponded to the initial load *F*_0_. This stress was held for 60 s, and the readings of the dial gauges were recorded on both sides as ε_0_ (left) and ε_0_ (right). Next, the load was continuously and evenly applied with a loading rate of 0.6 MPa/s. When the load was 1/3 of the ultimate compressive strength, the load was recorded as *F_n_* and held for 60 s, and the readings of the dial gauges are recorded as ε_n_ (left) and ε_n_ (right). The differences between the two values and their average did not exceed 20%; if it did, the load was be applied again (the unloading and loading speeds were consistent) until the test requirement was met. It should be noted that, in practice, the same batches of specimens were selected for the compression test in advance. The compression strength of these specimens was measured to convert the load corresponding to 0.5 MPa to eliminate the support displacement. The strength formed by the corresponding load was not necessarily 0.5 MPa corresponding to the test specimen, including 1/3 ultimate compressive strength.

Preloading started after the pretest was completed. Preloading started from the reference load *F*_0_ and was held for 60 s, and then the load was increased to *F*_n_, held for another 60 s, and again unloaded to the reference load *F*_0_. This preloading cycle was repeated three times.

The formal test was conducted as follows. After the last preloading cycle, starting from the reference load *F*_0_, the readings of the two dial gauges were recorded as ε_0_ (left) and ε_0_ (right) were recorded after the load was held for 60 s. The load was increased to *F_n_* and held for 60 s, and ε_n_ (left) and ε_n_ (right) were recorded. Afterward, the dial gauges and fixing frames were removed, the sample was loaded at a rate of 0.6 MPa/s until its failure, and the load during failure was recorded as *F_cp_*. If the difference between this *F_cp_* and that measured in the first group was more than 20%, this was marked. The compressive elasticity modulus of the concrete was processed based on the following formula:

(1) $$E_{c}=\frac{F_{n}-F_{0}}{A}\times \frac{L}{∆n}$$

where $E_{c}$ is the compressive elasticity modulus of the concrete (MPa); *F_n_* is the final load imposed in the elasticity modulus test (N, corresponding to 1/3 of *F_cp_*); *F*_0_ is the initial load imposed in the elasticity modulus test (N, corresponding to the load at 0.5 MPa); *L* is the length of the measurement gauge (mm); *A* is the loaded area of the sample (mm^2^); ∆n is the average of deformation differences on both sides of the sample under the influence of *F_n_* and *F*_0_ at the final loading (mm), i.e., ∆n=(ε_n_^(left)^+ε_n_^(right)^)/2−(ε_0_^(left)^+ε_0_^(right)^)/2; ε_0_ is the reading of the dial gauge when at a load of *F*_0_ (mm); and ε_n_ is the reading of the dial gauge at a load of *F_n_* (mm).

#### 2.3.3. Compression Test

This test was conducted for 3 days, 7 days, and 28 days; each duration was divided into early and late stages. The purpose of this test was to investigate the change in the compressive strength of the hybrid concrete during the starting and final stages when compared to ordinary concrete.

There were 6 groups of samples, including 3 groups of hybrid fiber–polymer concretes and 3 groups of C40 plain concrete, which corresponded to the three durations. There were 3 samples in each group, and thus 18 compressive samples in total, of which all had a size of 100 mm × 100 mm × 100 mm.

A fully automatic pressure testing machine in the laboratory that had a measuring range of 300 tons was used. The test was performed according to the Testing Methods of Cement and Concrete for Highway Engineering [28] and Test Methods Used for Steel Fiber Reinforced Concrete [29]. During the test, the regular, flat sides of the samples were selected as the compression surfaces, and the loading was under force control with a rate of 5 kN/s. The compressive strengths of the cubic hybrid fiber–polymer concrete samples were calculated using Equation (2). Since the size of the samples was non-standardized, the final compressive strength had to be multiplied by a factor of 0.95.

(2) $$f_{cu}=\frac{F}{A}$$

Here, $f_{cu}$ is the compressive strength of the concrete, *F* is the ultimate load (N), and *A* is the loaded area (mm^2^). The accuracy of the calculation was 0.01 MPa.

#### 2.3.4. Bending Test

The splitting tensile test and bending tensile test are indirect tensile tests. Like direct tensile tests, they can reflect the tensile properties of materials. A material’s structural design needs to consider the applicable scene and stress state of the material and the stress performance requirements of the structural pavement. This study used the bending tensile test to evaluate the tensile performance of the material under examination.

In order to eliminate the effect of shear stress, four point bending is a common test method. Its advantage is that the bending moment is evenly distributed and that the test results are therefore more accurate. In the test, the three-stage method is used to determine the loading position; that is, the two loading points at the loading position can divide the distance between the supports equally so as to determine the shear and bending spans of the specifications.

The bending test aimed at exploring whether the bending strength and crack resistance of the concrete were improved after the addition of the steel fiber, flexible fiber, and polymer emulsion, and if so, to what extent. The test was divided into ages of 3 days, 7 days, and 28 days to study the phase changes of strength and toughness for the hybrid concrete compared to that of ordinary concrete.

**Figure 4:**
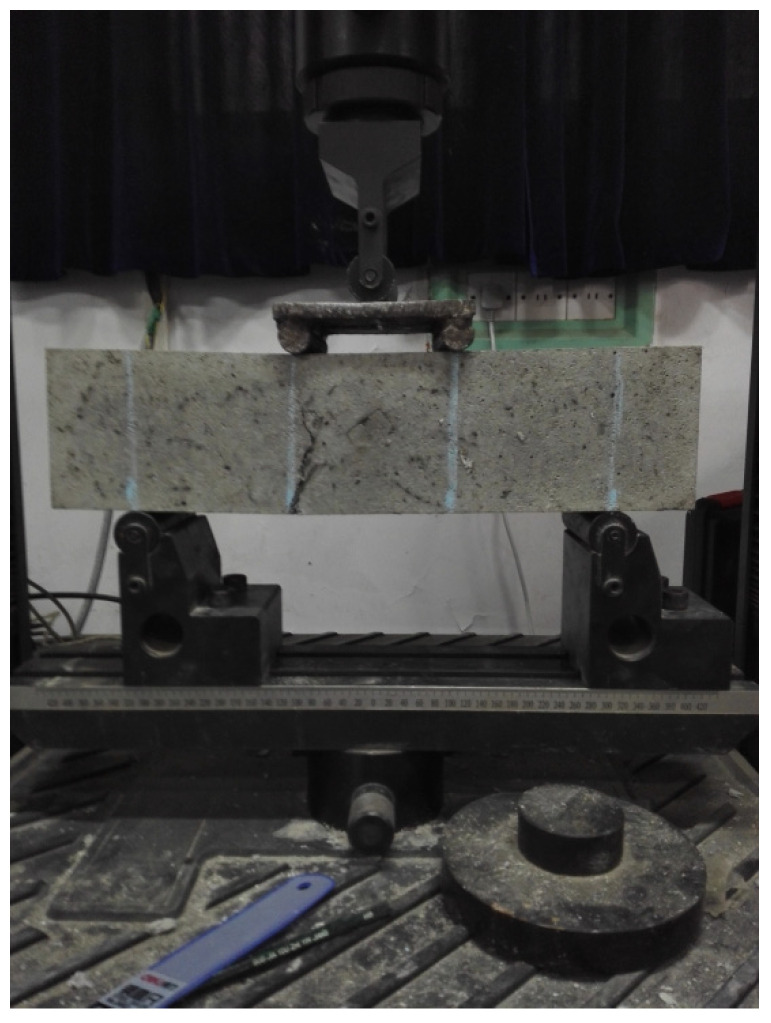
Schematic of four-point loading in the bending test.

The test was performed according to the *Testing Methods of Cement and Concrete for Highway Engineering* [28] and *Test Methods Used for Steel Fiber Reinforced Concrete* [29] by using the MTS universal testing machine from the laboratory, which possessed a loading range of 200 kN. As shown in Figure 4, the four-point bending test method was adopted. The distance between the bottom support point and the two ends of the sample was 5 cm, the distance between the two loading points at the top was 10 cm, and the distance between the loading point and the support point was 10 cm. The configured loading speed was 0.3 mm/min and was controlled by the displacement.

As in the compression test, there were 18 compressive samples in total, which included three groups of hybrid fiber–polymer concretes and 3 groups of C40 plain concretes, each containing 3 samples, though the sample size was 400 mm × 100 mm × 100 mm instead.

When the cross-section was within the pure bending section, the bending strength was calculated from the following formula:

(3) $$f_{f}=\frac{FL}{bh^{2}}$$

where $f_{f}$ is the bending strength (MPa), *F* is the ultimate load (N), *L* is the spacing between the supports (mm), *b* is the sample width (mm), and *h* is the height of the sample (mm).

Since the sample size (400 mm × 100 mm × 100 mm) was nonstandardized, the test result had to be multiplied by a factor of reduction of 0.85 according to the *Testing Methods of Cement and Concrete for Highway Engineering*.

#### 2.3.5. Sulfate Resistance

Under alternating high temperature and sulfate solution processing, shrinkage due to drying at high temperatures and expansion due to the erosion of sulfate solution cyclically occurred in the concrete. As the cycle repeated, the shrinkage and expansion of the concrete eventually led to extreme stress failure and internal cracks.

This test simulated the resistance capacities of the hybrid fiber–polymer concrete and plain concrete to natural sulfate attack, in contrast to the actual background of the industrially polluted environment.

This test was performed as per the *Standard for Test Methods of Long-term Performance and Durability of Ordinary Concrete* [30]. When the samples had been cured for 26 days, they were marked using a marking pen to avoid mixing the two kinds of concrete. Furthermore, they were transferred to an oven and baked at 80 °C for 48 h. Anhydrous sodium sulfate solution was mixed with water to obtain 10% sodium sulfate solution, and the samples were kept inside of this solution for the erosion test. After soaking for 15 h, the samples were taken out and put into the oven to bake at 48 °C for another 8 h. The above process constituted a single sulfate attack and high-temperature baking cycle. Fifteen cycles were performed before conducting the bending test, in which, again, the reduction coefficient was not considered.

For economical purposes, the sample size in this test was set to be 40 mm × 40 mm × 160 mm. A total of 12 samples were divided into two test groups and two control groups. The four-point loading mode was used in this case, though the supporting point was placed 20 mm away from the end of the sample, with a spacing of 40 mm between the loading points and a distance of 40 mm between the loading and the supporting point. The bending strength was calculated by using Equation (3), and the corrosion resistance was calculated by using:

(4) $$K_{f}=\frac{f_{cn}}{f_{c0}}\times 100$$

where $K_{f}$ is the corrosion resistance of the bending strength (%), $f_{cn}$ is the bending strength of the concrete sample noted after the sulfate resistance test (MPa), and $f_{c0}$ is the bending strength of the same-age concrete sample cured within room temperature (MPa).

#### 2.3.6. Electron Microscope Scanning

A scanning electron microscope (SEM) was used to determine the microstructural changes in ordinary concrete after the addition of polymer emulsion and steel fiber. The purpose of SEM was to explain the strength growth mechanism of the material and to explain how the fibers and concrete existed as a whole according to the observed microinterface between the fibers and concrete.

Focused electron beams were used by the SEM to scan and image the sample surface point by point. During the test, the cross-section of the sample was cleaned to avoid hampering of the observation results by concrete debris. Samples generally consisted of bulk materials or powder particles, and the imaging signals included secondary electrons, backscattered electrons, or absorbed electrons. The cross spot was taken as the electron source, and a micro electron beam with a certain energy, beam intensity, and beam spot diameter was formed because of the shrinking of the secondary condenser and the objective lens. Grid scanning was performed on the surface of the sample in a certain temporal and spatial sequence, driven by the scanning coil. Physical signals such as the secondary electron emission and the backscattered electrons were produced during the interaction of the focused electron beam with the sample. The amount of secondary electron emission changed along with the surface morphology of the sample. The detector collected the secondary electron signal and converted it into an electrical signal, which was fed into the kinescope grid after video amplification. A secondary electron image reflecting the surface morphology of the sample was obtained by modulating the brightness of the kinescope that was scanned synchronously with the incident electron beam.

## 3. Results and Discussions

### 3.1. Basic Mechanical Properties

#### 3.1.1. Slump

The slump test was conducted thrice and was categorized as groups A, B, and C. Each group contained the the same mix proportion, but for more accurate data, the test was conducted three times, and the average value was taken. The measured data were collected immediately after the slump barrel was lifted and after intervals of 0.5 h, 1.0 h, 1.5 h, and 2.0 h. Table 2 shows the calculated slump values.

If hybrid fiber–polymer concrete were applied to pavement materials, to facilitate construction, it would be expected that it had good fluidity at the beginning and good plasticity after forming. Therefore, this paper tested the variation law of slump with time to examine the hybrid concrete’s working performance.

- Test result analysis

The three groups of test data showed very low discreteness (Table 2). The concrete fluidity was relatively good within the 0–1 h interval, indicating strong peaceability of the hybrid fiber–polymer concrete at an early stage. The concrete fluidity was relatively stable from the period of 1 h to 2 h, implying that the overall cohesion was high. The results were good, which confirmed that the hybrid fiber–polymer concrete under the proposed mix proportion showed a reliable working performance.

#### 3.1.2. Elasticity Modulus Test

- Test result

In the elasticity modulus loading test, F_0_ = 11.25 kN, F_n_ = 332.1 kN. By feeding these data into Equation (1), the elasticity moduli A, B, and C were found to be 36.41 GPa, 32.63 GPa, and 38.75 GPa. Based on the results presented above, the elasticity modulus of the hybrid fiber–polymer concrete was found to be 35.93 GPa.

- Test result analysis

From the test results, the elastic modulus of the hybrid fiber–polymer concrete was slightly higher than that of ordinary cement concrete, indicating that the hybrid material had high resistance to deformation. If it were applied to steel deck pavement, it would significantly increase the stiffness of the structure compared with the commonly used asphalt concrete.

#### 3.1.3. Compression Test

- Test result

**Figure 5:**
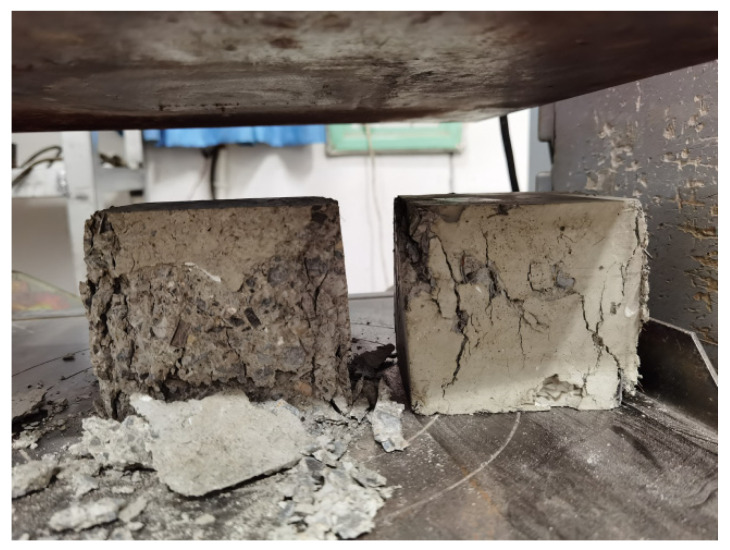
Comparison of compression failures in the plain concrete (left) and hybrid fiber–polymer concrete (right).

Table 3 summarizes the compressive strengths after 3, 7, and 28 days, and a comparison of compressive failures in the hybrid fiber–polymer concrete and plain concrete is shown in Figure 5.

- Test result analysis

As shown in Table 3, it was found that the compressive strength of the hybrid fiber–polymer concrete was much higher than that of the ordinary concrete, with an increase of 31.23% over the 28-day curing period.

According to the characteristics of failure displayed in Figure 5, the hybrid fiber–polymer concrete had a plastic flattening state, while the ordinary concrete demonstrated brittle crushing. Also, after the compression failure, the height of the hybrid fiber–polymer concrete sample was significantly decreased compared to the plain concrete, and its transverse width was wider. The addition of fiber and polymer significantly improved the failure characteristics of concrete, which showed significant plastic failure. This failure characteristic gives the hybrid fiber–polymer concrete studied in this paper certain advantages in terms of safety of compression failure.

#### 3.1.4. Bending Test

- Test result

**Figure 6:**
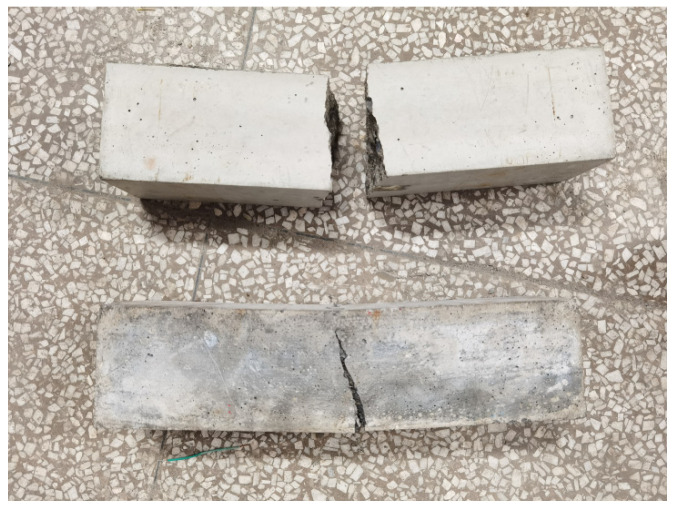
Failure modes of the plain concrete and hybrid fiber–polymer concrete.

**Figure 7:**
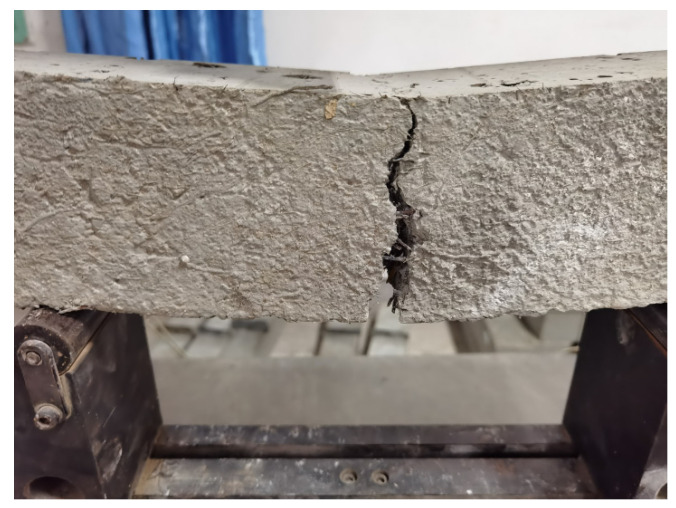
Details of crack resistance by the hybrid fiber–polymer concrete.

Table 4 summarizes the bending strengths after 3, 7, and 28 days, while Figure 6 and Figure 7 compare the bending failures of the hybrid fiber–polymer concrete and plain concretes.

- Test result analysis

Comparative analysis of the bending test results displayed in Table 4 showed that the bending strength of the hybrid fiber–polymer concrete was always higher than that of the ordinary concrete. On day 28, the former possessed strength 3.15 times that of the latter, with a rapid increase during the initial stage and gentle growth during the later stages. Here, the initial stage refers to several days after forming, and the later stage refers to the period from a few days after the end of the early stage to 28 days.

According to the bending failure patterns revealed in Figure 6 and Figure 7, the hybrid fiber–polymer concrete did not break off because of the tightly held parts tied together by the fiber and the polymer emulsion, reducing the secondary damage caused to the whole structure. Ordinary concrete, however, showed brittle fractures after bending failure.

In the process of preliminary exploration and research, it was found that greater numbers of steel fibers in the cross-section corresponded to a stronger bending capacity. When the total number of steel fibers was fixed, enhancing the uniform dispersion and reducing the agglomeration of the fibers effectively increased the number of steel fibers in the cross-section. As for the distribution, the bending capacity was the highest when the steel fiber in the cross-section was distributed vertically relative to the fractured surface. To a certain extent, improving the uniform dispersion of the steel fibers also facilitated optimal distribution. The force of bonding between the steel fiber and the concrete matrix depends on the cementation of the cement and the polymer emulsion colloids. The choice of cement and the types of polymer emulsion that best suit the steel fiber could further improve the bending capacity of the hybrid fiber–polymer concrete.

**Figure 8:**
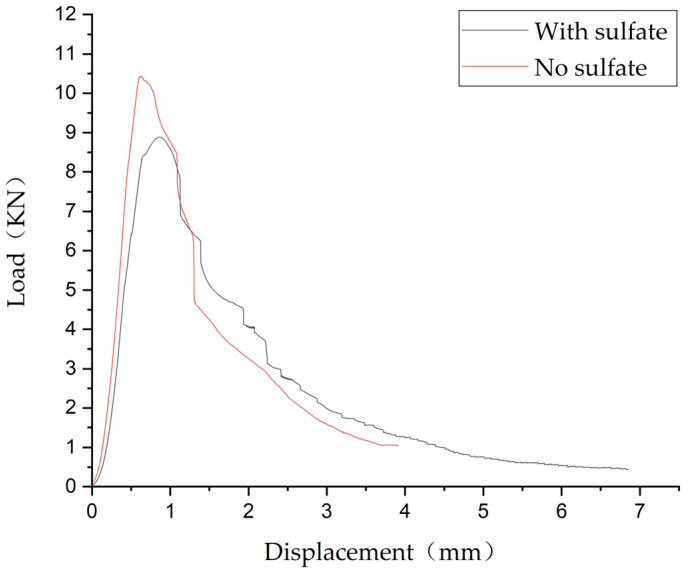
The load–displacement curves for the hybrid fiber–polymer concrete with and without sulfate attack.

Figure 8 shows the load displacement curve of the specimen under a bending test. The specimen still has the ability to maintain the load after reaching the ultimate load, and its displacement continued to increase. The curve had a long, flat section after reaching the maximum load, which shows that the specimen had typical plastic failure characteristics and still had significant load holding capacity after reaching the maximum load. This would be conducive to the safety and durability of structures composed with this material in engineering practice.

### 3.2. Sulfate Resistance

- Test result

The load–displacement curves for the hybrid fiber–polymer concrete with and without sulfate attack were compared, as shown in Figure 8. Table 5 summarizes the bending strengths of the test groups and the control groups.

Table 6 summarizes the test group and control group strengths and corrosion resistances given below.

- Test result analysis

The bending test results of the two materials cured with and without sulfate attack (Table 5 and Table 6, respectively) were compared, and it was found that the corrosion resistance of the hybrid fiber–polymer concrete and the ordinary concrete reached 81.31% and 83.45%, respectively, indicating that the hybrid fiber–polymer concrete had the same corrosion resistance as ordinary concrete.

### 3.3. Structural Characteristics of the Hybrid Fiber–Polymer Concrete under Electron Microscopy

- Test result and analysis

**Figure 9:**
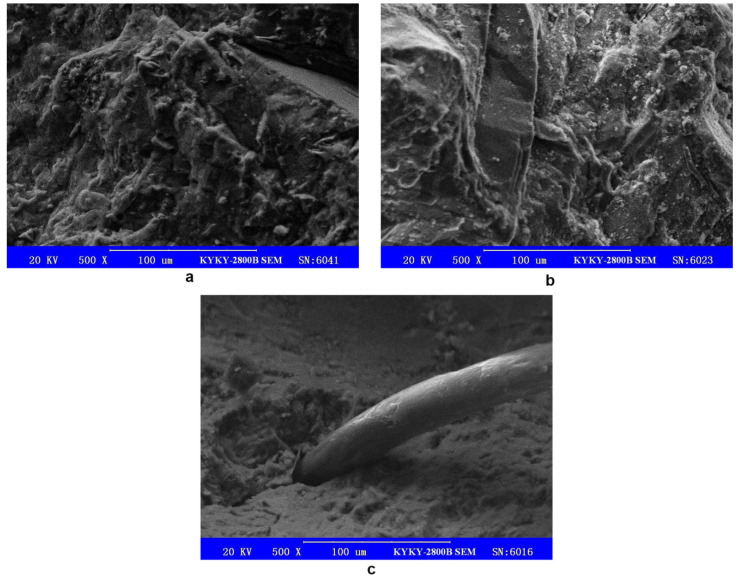
Cross-sectional SEM images: (**a**)**,** surface of the hybrid fiber–polymer concrete; (**b**), surface of plain concrete; (**c**), connection between steel fiber and concrete matrix.

The SEM magnification was adjusted to 500×, and the resulting cross-sectional SEM images of the plain concrete and the hybrid fiber–polymer concrete are shown in Figure 9.

From Figure 9a,b, it can be seen that many microcracks and small pits were present on the surface of the ordinary concrete, while on the surface of the hybrid fiber–polymer concrete, a layer of dense cementing polymer colloid filled a few microcracks and pits, making the whole concrete seem more compact. The hybrid fiber–polymer concrete had fewer microholes and microcracks than the ordinary concrete and thus appeared to be more integrated. This feature might have allowed the hybrid fiber–polymer concrete to show strength and corrosion resistance higher than the ordinary concrete.

Figure 9c shows the connection between a steel fiber and the concrete matrix. The steel fiber and concrete were integrated by wrapping steel fiber with slurry; said slurry was a polymer–cement net formed by polymer latex and cement. It can be inferred that the adhesion between polymer–cement paste and steel fiber was an important factor in the hybrid material. However, the slurry did not completely wrap around the steel fiber. The contact area between the steel fiber and the cement paste directly affects the binding force between the steel fiber and the cement paste. Future research can improve the contact area between the steel fiber and the cement paste by adjusting the mix proportion or improving the workability of the cement paste.

## 4. Conclusions

In this study, hybrid fiber composed of sheared wavy steel fiber, ultrashort ultrafine steel fiber, and polypropylene fiber was utilized to play a part in the crack resisting and reinforcing effect of fiber materials. In addition, polymer was added to improve the deformability of the concrete. Through research conducted on the basic mechanical properties and environmental durability of the hybrid fiber–polymer concrete, the following conclusions were drawn.

- In the slump test, the hybrid fiber–polymer concrete showed good water retention, fluidity, and cohesion, which met actual engineering requirements. The hybrid concrete had an average modulus of elasticity of 35.93 GPa, the material stiffness of which was equivalent to that of ordinary cement concrete.
- The compression test showed that the compressive strengths of the ordinary concrete on days 3 and 7 were slightly higher than those of the hybrid fiber–polymer concrete. In the 28-day curing case, however, the strength of the hybrid fiber–polymer concrete was significantly higher than that of ordinary concrete, with an increase of 31.23%.
- The bending test revealed that the bending strength of the hybrid fiber–polymer concrete was significantly higher than that of the ordinary concrete during both stages, demonstrating a rapid increase during the early stage and a gentle growth during the later stage. As for the failure mode, the hybrid fiber–polymer concrete did not break off, as the parts were tightly tied together by the fiber and the polymer emulsion, which would reduce the threat of secondary failure of the whole structure in practice. However, the ordinary concrete underwent brittle fracture after bending failure.
- In the bending test under sulfate attack, the corrosion resistance of the hybrid fiber–polymer concrete was 81.31%. The curve after the peak load indicated that the steel fiber still played a role in resisting bending, indicating that soaking in sulfate solution did not separate the steel fiber from the concrete matrix. Although the bending strength was slightly reduced, the corrosion resistance (81.31%) of the hybrid fiber–polymer concrete revealed favorable adaptability to sulfate environments.

The SEM test demonstrated that the hybrid fiber–polymer concrete was more integrated than ordinary concrete, as it had fewer microholes and microcracks when compared with ordinary concrete. This characteristic could lead to its higher bending and compressive strengths when compared to those of ordinary concrete. SEM showed that the steel fiber and concrete were integrated by wrapping the steel fiber with a slurry, and that the slurry was a polymer–cement net formed by polymer latex and cement. 

## Figures and Tables

**Table 1:** Primary mechanical properties of different types of steel fibers.

Type	Length (mm)	Diameter (mm)	Length to Diameter Ratio	Bending Performance	Elasticity Modulus (GPa)	Tensile Strength (MPa)
Wavy steel fiber	35	0.87	40	>90	210	425
Ultrashort ultrafine steel fiber	6	0.2	30	>90	240	880
Polypropylene flexible fiber	12	0.03	400	>90	3.85	500

**Table 2:** Slump values of the hybrid fiber–polymer concrete at different moments.

Time (h)	0	0.5	1.0	1.5	2.0
Slump A (mm)	94	100	105	105	105
Slump B (mm)	90	98	103	104	105
Slump C (mm)	93	101	104	104	104

**Table 3:** Compressive strengths of samples at different ages (hybrid concrete refers to the high-content hybrid fiber–polymer concrete studied in this paper; plain concrete refers to ordinary concrete C40 without fiber or polymer).

Time	Type	Load (kN)	Strength after Reduction (MPa)	Average Strength (MPa)
3 days	Plain concrete	211.99	20.14	19.92
		223.99	21.28	
		192.99	18.34	
	Hybrid concrete	213.99	20.33	20.30
		218.99	20.81	
		207.99	19.76	
7 days	Plain concrete	370.99	35.25	39.36
		416.99	39.62	
		454.99	43.23	
	Hybrid concrete	350.99	33.35	35.28
		391.99	37.24	
		370.99	35.25	
28 days	Plain concrete	422.99	40.19	40.25
		445.99	42.37	
		401.99	38.19	
	Hybrid concrete	559.98	53.20	52.82
		556.98	52.92	
		550.98	52.35	

**Table 4:** Bending strengths of samples during different ages.

Time	Type	Load (kN)	Strength after Reduction (MPa)	Average Strength (MPa)
3 days	Plain concrete	9.37	2.39	2.42
		9.03	2.30	
		10.07	2.57	
	Hybrid concrete	27.34	6.97	6.97
		17.16	4.37 *	
		27.84	7.10	
7 days	Plain concrete	13.50	3.44	3.60
		13.70	3.49	
		15.17	3.87	
	Hybrid concrete	28.74	7.33	7.61
		28.81	7.34	
		31.97	8.15	
28 days	Plain concrete	12.86	3.88	3.95
		15.95	4.07	
		14.11	3.9	
	Hybrid concrete	44.50	11.35	11.51
		45.20	11.53	
		45.70	11.65	

**Note:** * indicates that the abnormal data were ignored when calculating the average value.

**Table 5:** Test results of the bending strength of each sample.

Type	Condition	Load (kN)	Strength (MPa)	Average Strength (MPa)
Hybrid concrete	No sulfate	10.43	19.55	18.08
	No sulfate	9.70	18.19	
	No sulfate	8.81	16.51	
	With sulfate	8.89	16.66	14.70
	With sulfate	7.60	14.25	
	With sulfate	7.04	13.20	
Plain concrete	No sulfate	3.89	7.30	7.13
	No sulfate	3.84	7.21	
	No sulfate	3.67	6.87	
	With sulfate	3.26	6.11	5.95
	With sulfate	3.25	6.09	
	With sulfate	3.00	5.63	

**Table 6:** Test results of the bending strength of each sample after sulfate attack.

Type	Condition	Strength (MPa)	Corrosion Resistance (%)
Hybrid concrete	Test group	14.70	81.31
	Control group	18.08	
Plain concrete	Test group	5.95	83.45
	Control group	7.13	

## Data Availability

The data used to support the findings of this study are included within the article.

## Abbreviation

SEM, scanning electron microscope.

## References

[1] Beeldens A., Van Gemert D., Schorn H., Ohama Y., Czarnecki L. (2005). From microstructure to macrostructure: An integrated model of structure formation in polymer-modified concrete. Mater. Struct..

[2] Göbel L., Bos C., Schwaiger R., Flohr A., Osburg A. (2018). Micromechanics-based investigation of the elastic properties of polymer-modified cementitious materials using nanoindentation and semi-analytical modeling. Cement. Concr. Compo..

[3] Lee S.K., Jeon M.J., Cha S.-S., Park C.G. (2017). Mechanical and permeability characteristics of latex-modified fiber-reinforced roller-compacted rapid-hardening-cement concrete for pavement repair. Appl. Sci..

[4] Toufigh V., Hosseinali M., Shirkhorshidi S.M. (2016). Experimental study and constitutive modeling of polymer concrete’s behavior in compression. Constr. Build. Mater..

[5] Zhang X.L., Cao H., Guo X.H. (2013). Study on Compressive Stress-Strain Relationship of Polymer-Modified Concrete. Adv. Mater. Res..

[6] Rezakhani R., Scott D.A., Bousikhane F., Pathirage M., Moser R.D., Green B.H., Cusatis G. (2021). Influence of steel fiber size, shape, and strength on the quasi-static properties of ultra-high performance concrete: Experimental investigation and numerical modeling. Constr. Build. Mater..

[7] Özkılıç Y.O., Aksoylu C., Arslan M.H. (2021). Experimental and numerical investigations of steel fiber reinforced concrete dapped-end purlins. J. Build. Eng..

[8] Vita N., Sharma A. (2021). Behaviour of single bonded anchors in non-cracked and cracked steel fiber reinforced concrete under short-time tensile loading. Eng. Struct..

[9] Wei M., Liu Y., Tang L., Huang X. (2009). Experimental Study on Fiber Reinforced Concrete. Key Eng. Mater..

[10] Yoo D.Y., Yoon Y.S., Banthia N. (2015). Flexural response of steel-fiber-reinforced concrete beams: Effects of strength, fiber content, and strain-rate. Cem. Concr. Compos..

[11] Yuan H., Li Y. (2015). The Combined Effects of Fiber and Cement-Based Composite Materials. Appl. Mech. Mater..

[12] Meng G., Gao B., Zhou J., Cao G., Zhang Q. (2016). Experimental investigation of the mechanical behavior of the steel fiber reinforced concrete tunnel segment. Constr. Build. Mater..

[13] Barton N.R. Investigation, design and support of major road tunnels in jointed rock using NMT principles [C]. Proceedings of the IX Australian Tunnelling Conference.

[14] Buratti N., Ferracuti B., Savoia M. (2013). Concrete crack reduction in tunnel linings by steel fibre-reinforced concretes. Constr. Build. Mater..

[15] Kytinou V.K., Chalioris C.E., Karayannis C.G., Elenas A. (2020). Effect of Steel Fibers on the Hysteretic Performance of Concrete Beams with Steel Reinforcement—Tests and Analysis. Materials.

[16] Hung C.-C., Lee H.S., Si N.C. (2019). Tension-stiffening effect in steel-reinforced UHPC composites: Constitutive model and effects of steel fibers, loading patterns, and rebar sizes. Compos. Part B Eng..

[17] Carneiro J.A., Lima P.R.L., Leite M.B., Toledo Filho R.D. (2014). Compressive stress–strain behavior of steel fiber reinforced-recycled aggregate concrete. Cement Concr. Compo..

[18] Gao D., Wang F. (2021). Effects of recycled fine aggregate and steel fiber on compressive and splitting tensile properties of concrete. J. Build. Eng..

[19] Gholamhoseini A., Khanlou A., MacRae G., Scott A., Hicks S., Leon R. (2016). An experimental study on strength and serviceability of reinforced and steel fibre reinforced concrete (SFRC) continuous composite slabs. Eng. Struct..

[20] Oliveira Júnior L.Á.D., Borges V.E.D.S., Danin A.R., Machado D.V.R., Araújo D.D.L., El Debs M.K., Rodrigues P.F. (2010). Stress-strain curves for steel fiber-reinforced concrete in compression. Matéria.

[21] Alsaif A., Garcia R., Figueiredo F.P., Neocleous K., Christofe A., Guadagnini M., Pilakoutas K. (2019). Fatigue performance of flexible steel fibre reinforced rubberised concrete pavements. Eng. Struct..

[22] Zaki R.A., AbdelAleem B.H., Hassan A.A., Colbourne B. (2021). Impact resistance of steel fiber reinforced concrete in cold temperatures. Cement Concr. Compo..

[23] Zhang L., Liu J., Liu J., Zhang Q., Han F. (2018). Effect of steel fiber on flexural toughness and fracture mechanics behavior of ultrahigh-performance concrete with coarse aggregate. J. Mater. Civ. Eng..

[24] Zhang S., Zhang C., Liao L. (2019). Investigation on the relationship between the steel fibre distribution and the post-cracking behaviour of SFRC. Constr. Build. Mater..

[25] Cao H., Zhang X.L., Guo X.H., Feng J.J. (2014). Experimental Study on Anti-Penetration Performance of Steel Fiber Reinforced Polymer-Modified Concrete. Adv. Mater. Res..

[26] Ministry of Housing and Urban Rural Development of the People’s Republic of China (2016). Standard for Test Method of Performance on Ordinary Fresh Concrete.

[27] Ministry of Construction of the People’s Republic of China (2003). Standard for Test Method of Mechanical Properties on Ordinary Concrete.

[28] Ministry of Transport of the People’s Republic of China (2020). Testing Methods of Cement and Concrete for Highway Engineering.

[29] China Engineering Construction Standardization Association (1991). Test Methods Used for Steel Fiber Reinforced Concrete.

[30] Ministry of Housing and Urban Rural Development of the People’s Republic of China (2009). Standard for Test Methods of Long-Term Performance and Durability of Ordinary Concrete.

